# Perinatal Bisphenol S Exposure Induces Hepatotoxicity in Female Rat Offspring: Insights into Disrupted Hepatic Cholesterol Homeostasis

**DOI:** 10.3390/toxics14100878

**Published:** 2026-09-30

**Authors:** Hongming Huang, Jingyi Zhang, Jing Jin, Ying Xie, Rui Pan, Min Zhao, Biran Zhu, Jianghuan Hua

**Affiliations:** 1 School of Basic Medical Sciences, Hubei University of Chinese Medicine, Wuhan 430065, China; 2431601091@stmail.hbucm.edu.cn (H.H.); xying23@stmail.hbucm.edu.cn (Y.X.); zmin13@hbucm.edu.cn (M.Z.); 2 Department of Pediatrics, Institute of Pediatric Diseases and Rare Diseases, Xiangyang Central Hospital, Affiliated Hospital of Hubei University of Arts and Science, Xiangyang 441021, China; zjy_ftg@163.com (J.Z.); prdoctor1979@163.com (R.P.); 3 Hubei Shizhen Laboratory, Wuhan 430061, China; venajin@163.com; 4 The First Clinical Medical School, Hubei University of Chinese Medicine, Wuhan 430065, China; 5 Hubei Provincial Hospital of Traditional Chinese Medicine, Wuhan 430061, China

**Keywords:** Bisphenol S, perinatal exposure, hepatotoxicity, bile acid synthesis, female offspring

## Abstract

Recent research has highlighted concerns regarding the hepatotoxicity of Bisphenol S (BPS), particularly its potential transgenerational effects. In this study, we investigated the effects of perinatal BPS exposure on female offspring (F1) by exposing pregnant Sprague-Dawley (SD) rats (F0) to 0.05 and 20 mg/kg BPS from gestational day 6 to postnatal day 21. Results showed that perinatal BPS exposure resulted in increased body weight, higher liver coefficient, and impaired liver function in female rat offspring (F1). Untargeted metabolomics analysis revealed suppression of the primary bile acid biosynthesis pathway, and network toxicology predicted cholesterol metabolism and bile secretion as key pathways, suggesting altered cholesterol and bile acid homeostasis in the liver of female offspring. Validation studies demonstrated activation of cholesterol synthesis, as shown by upregulation of sterol regulatory element-binding protein 2 (SREBP2), squalene epoxidase (SQLE), and 3-hydroxy-3-methylglutaryl-CoA reductase (HMGCR). Furthermore, the down-regulated expression levels of acetyl-CoA acetyltransferase 1 (ACAT1), ATP-binding cassette sub-family G member 8 (ABCG8), and ATP-binding cassette sub-family G member 5 (ABCG5) indicated that pathways associated with cholesterol conversion, including cholesterol esterification, transport, and secretion as very low-density lipoprotein cholesterol (VLDL-C), were suppressed. Specifically, reduced transcription of ACAT1, ABCG8, and ABCG5, which are involved in cholesterol esterification and cholesterol efflux, was observed. This imbalance was associated with hepatic cholesterol accumulation and hepatotoxicity, although whether cholesterol accumulation was a cause or a consequence of liver injury remained unclear. In summary, our findings demonstrate that perinatal BPS exposure disrupted hepatic cholesterol homeostasis, which was associated with cholesterol accumulation and hepatotoxicity in F1 female rats.

## 1. Introduction

Bisphenol S (BPS) serves as a substitute for the commonly utilized plastic additive, Bisphenol A (BPA) [1,2]. It is utilized in various packaging industries, such as maternal and childcare products, thermal papers, and canned foods [3,4]. The increased use of BPS raises its potential for environmental release. For instance, in indoor dust specimens, the mean concentration of BPS in China was found to be 0.63 μg/g [5]. Surface water samples have also been reported to be considerably affected by BPS contamination, with concentrations reaching 0.006 μg/L in Taihu Lake, China [6], and as high as 7.2 μg/L in Adyar River, India [7]. Furthermore, BPS has been detected in diverse food products [8,9], indicating potential human exposure. Recent biomonitoring studies have reported widespread BPS exposure in the general population, including pregnant women. In the Yangtze River Basin, China, serum BPS concentrations among general residents (*n* = 240) averaged 1.43 ng/mL, while urinary BPS was detected in 62–68% of pregnant women (*n* = 845) from the same region, with median concentrations reaching 0.07 ng/mL and maximum levels up to 1.77 ng/mL [10]. In the United States, BPS was detected in 27% of urine samples from a Boston cohort [11]. In Europe, a Dutch cohort study found higher median concentrations of BPS (0.36 μg/L) among 1396 pregnant women [12], indicating geographical variability in exposure levels. Importantly, BPS can cross the placental barrier and may accumulate in fetal tissues [13], raising concerns about its developmental toxicity. A population-based prospective cohort demonstrated that elevated BPS levels in maternal urine have been associated with adverse birth outcomes, including larger fetal head circumference and higher fetal weight, as well as a lower risk of being born small for gestational age [14]. Although these findings suggest a potential growth-promoting effect, such alterations in fetal growth trajectories may reflect underlying endocrine and metabolic disruption, with potential long-term consequences for offspring health.

BPS exhibits multiple toxic effects, including reproductive toxicity [15,16], neurotoxicity [17,18], developmental toxicity [19,20], and endocrine disruption [21,22]. The liver, an essential organ for metabolism and highly responsive to xenobiotics, is also susceptible to BPS. For example, exposure to 5–5000 μg/kg BPS over 8 weeks has been reported to cause localized necrosis and lymphocyte infiltration in the liver tissue of male mice [23]. In addition, post-weaning exposure to 25, 50, and 100 μg/kg BPS for 10 weeks has been shown to induce significant liver damage, characterized by varying degrees of necrosis and steatosis [24]. In a previous study, exposing pregnant rats to BPS (0.05 and 20 mg/kg) adversely affected the development and fertility of their offspring [16]. Chronic exposure to low-dose BPS (50 and 500 μg/kg/day for 20 weeks) altered hepatic lipid synthesis and mitochondrial function in male Wistar rats, with increased serum triglycerides and LDL-cholesterol, and dysregulated antioxidant enzymes [25]. Pal et al. [26] reported that BPS exposure at 30–120 mg/kg/day for 30 days impaired blood functions and induced cardiovascular risks in male rats, with elevated serum total cholesterol, triglycerides, LDL, VLDL, AST, ALT, and ALP. Meng et al. [27] found that perinatal exposure to 100 ng/g BPS induced severe steatosis in the liver of male mouse offspring. Although bisphenol analogs are known to exert hepatotoxic effects through mechanisms such as oxidative stress, inflammation, and lipid metabolism disruption [28], the impact of perinatal BPS exposure on hepatic cholesterol homeostasis in female offspring remains unexplored. Furthermore, the potential role of cholesterol metabolism, a central pathway linking hepatic function to lipid homeostasis, has not been explored in this context. Cholesterol metabolism plays a central role in maintaining hepatic homeostasis, and its dysregulation, particularly the accumulation of free cholesterol, has been implicated in the pathogenesis of liver injury, including steatosis, inflammation, and fibrosis [29,30]. However, whether perinatal BPS exposure disrupts hepatic cholesterol metabolism in female offspring remains unknown.

In the present study, we exposed perinatal Sprague-Dawley (SD) rats to environmentally relevant levels of BPS and employed an integrated approach combining untargeted metabolomics, network toxicology, and molecular validation to: (1) assess its hepatotoxic effects on female offspring, and (2) elucidate the underlying mechanisms involving cholesterol synthesis, conversion, and efflux pathways. Our findings aim to advance the understanding of BPS-induced hepatotoxicity in female offspring and provide critical data for environmental health risk assessment of BPS as a BPA substitute. This study extends previous male-focused or single-endpoint studies by focusing on female offspring and defining a multi-step cholesterol-related mechanism.

## 2. Materials and Methods

### 2.1. Chemicals and Reagents

BPS (CAS: 80-09-1; >98%) and urethane (CAS: 51-79-6; ≥98.0%) were obtained from Shanghai Yuanye Bio-technology Co., Ltd (Shanghai, China). All other chemicals were of analytical grade (Table S1). BPS was dissolved in 0.1% anhydrous ethanol (*v*/*v*) to prepare stock solutions. Working solutions were freshly prepared daily by diluting the stock solution with vehicle, and all solutions were stored in amber glass vials to prevent photodegradation.

### 2.2. Animal Experiments

Forty-two healthy adult SPF-grade SD rats [30 females (200–250 g) and 12 males (280–300 g)] were used in this study [certificate NO. SYXK (Hubei) 2023-0067]. The rats were housed at the Hubei Traditional Chinese Medicine Rat Animal Experimental Center. They were kept under a 12 h light-dark cycle, with ambient humidity ranging from 40–70% and an ambient temperature of 23°C ± 2°C. The animals were provided with unrestricted access to a standard specific pathogen-free (SPF)-grade rat chow (Beijing Keao Xieli Feed Co., Ltd., Beijing, China; catalog no. 22023113) and water. All animals were provided with nesting material and polycarbonate shelters as environmental enrichment and the experiments commenced following a 1-week acclimation period (i.e., acclimatization). Gestation day 0 (GD0) was defined as the first day of pregnancy. Non-pregnant females and all males were then excluded from subsequent experiments. Oral gavage was selected because dietary intake is the predominant route of human BPS exposure [31]. Pregnant rats received daily oral gavage from GD6 to postnatal day 21 (PND21), and were randomly assigned to three groups: Control (Con: Equal volume of vehicle), Low-dose BPS (BPS-L: 0.05 mg/kg body weight/day), and High-dose BPS (BPS-H: 20 mg/kg body weight/day), with six dams per group. The total volume of administered solution was kept constant across all groups. Three replicate cages (each containing 2 female rats) were used for each exposure treatment. All groups received 0.1% ethanol (*v*/*v*) as the vehicle. BPS exposure doses were selected based on the U.S. Environmental Protection Agency (EPA) reference dose for BPA (0.05 mg/kg/day), as no official RfD exists for BPS, and with reference to previous studies reporting that comparable dose levels induced reproductive and transgenerational toxicity [16] as well as hepatic lipid accumulation and metabolic disruption [27] in perinatally exposed offspring. After PND21, female offspring were housed in separate cages, with BPS-free drinking water in glass bottles to prevent further BPS exposure. To avoid litter effects, no more than one female offspring was selected from each litter for any given endpoint, ensuring that each biological replicate originated from a distinct litter. For each endpoint, one female offspring was randomly selected from each litter, and all data presented represent values from individual animals, with litter as the independent experimental unit (*n* = 3–6 per group). Specifically, *n* = 6 litters/group were used for growth and body weight measurements; *n* = 3 litters/group for histology; *n* = 4 litters/group for metabolomics; and *n* = 3/6 litters/group for quantitative real-time PCR (qRT-PCR) and Western blotting.

**Figure 1:**
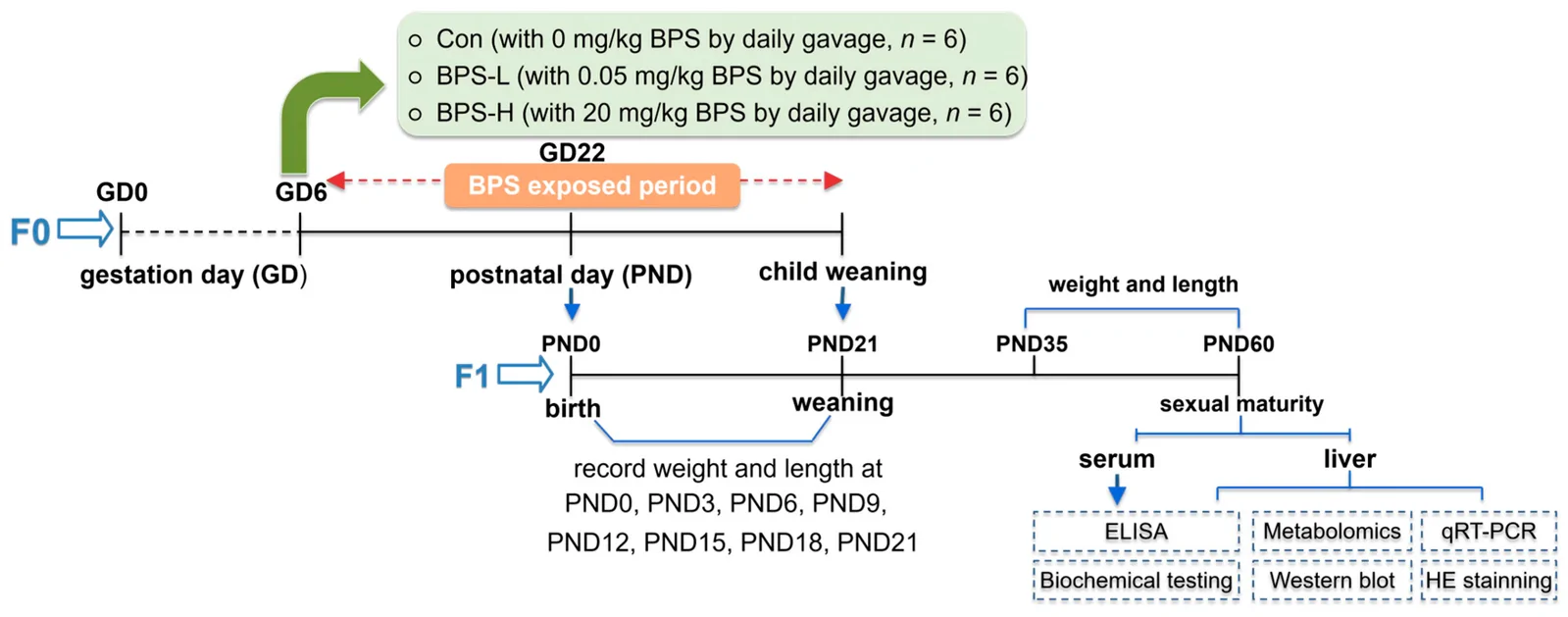
Experimental design. Pregnant rats were administered BPS by oral gavage from GD6 to PND21 at doses of 0.05 or 20 mg/kg body weight/day (dissolved in vehicle containing 0.1% ethanol, *v*/*v*), with a vehicle control group receiving 0.1% ethanol alone. The effects on the structure and function of the liver of the offspring (F1) of female rats were investigated at PND60. GD, gestation day; PND, postnatal day.

Body weight and length of female offspring (F1) were measured every three days before weaning and again on PND35 and PND60. GD6–PND21 covered the perinatal exposure window, and PND60 corresponded to sexual maturity for assessing persistent post-weaning effects. At the end of the experiment, rats were anesthetized by intraperitoneal injection of 1.2 g/kg urethane (20% *v*/*v*). Serum samples were collected from F1 females for enzyme-linked immunosorbent assay (ELISA) and biochemical analyses. One liver lobe was used for hematoxylin–eosin (H&E) staining, and the remaining hepatic tissue was flash-frozen in liquid nitrogen and stored at −80 °C for subsequent molecular biology analyses. The experimental workflow is illustrated in Figure 1.

Animals were monitored daily for signs of distress, pain, or weight loss. Humane endpoints included >20% body weight loss, inability to access food or water, or moribund condition. No adverse events were observed during the study. All procedures complied with the U.K. Animals (Scientific Procedures) Act 1986, EU Directive 2010/63/EU, and the National Research Council’s Guide for the Care and Use of Laboratory Animals. All animal experiments were conducted with the approval of the Experimental Animal Ethics Committee of Hubei University of Chinese Medicine (No. HUCMS 00309450). The completed ARRIVE Essential 10 Compliance Questionnaire has been submitted.

### 2.3. Histopathological Analyses and Biochemical Measurements

Following the protocol reported by Luo et al. [32], H&E staining was performed on liver samples from F1 female offspring (N = 3). The detailed procedure is provided in Text S1, and liver function indicators were assessed using commercial kits (Table S1). Protein content was normalized for all parameters using the BCA protein quantification assay.

### 2.4. Untargeted Metabolomics

Metabolites were extracted from liver samples of each female offspring (*n* = 4) and analyzed using ultra-high-performance liquid chromatography-mass spectrometry (UHPLC-MS) at Beijing Biomarker Technologies Co., Ltd. BMKCloud (Beijing, China). In brief, metabolites were extracted with an acetonitrile-water (1:1) solvent system, followed by vacuum drying and resuspension. LC-MS detection was performed on a Thermo Ultim3000 High-Performance Liquid Chromatograph (Thermo Fisher Scientific, Waltham, MA, USA) coupled with a Thermo Orbitrap Exploris 480 High-Resolution Mass Spectrometer (Thermo Fisher Scientific, Waltham, MA, USA) using a Waters Acquity UPLC HSS T3 column (Waters Corporation, Milford, MA, USA). Data were acquired in both positive and negative ion modes with data-dependent MS/MS. Peak alignment, extraction, and quantification were performed, and metabolites were identified by comparison with the mzCloud, MZvault, and ChemSpider databases. Differential metabolites were screened based on VIP > 1, |log2FC| > 1, and *p* < 0.05. KEGG pathway enrichment analysis of the differential metabolites was performed using the BMKCloud platform, and pathways with *p* < 0.05 or FDR < 0.05 were considered significantly enriched. Data analysis and visualization were conducted on the BMKCloud platform (www.biocloud.net) provided by Beijing Biomarker Technologies Co., Ltd (Beijing, China). The detailed procedure, including reagents, instruments, chromatographic and mass spectrometric parameters, is provided in Text S2.

### 2.5. Network Toxicology

BPS-related targets were obtained from SEA (v4.0; https://sea.bkslab.org/), TargetNet (http://targetnet.scbdd.com/), CTD (2025 update; https://ctdbase.org), Swiss Target Prediction (Release 2024; http://swisstargetprediction.ch/), and STITCH (v5.0; https://www.ebi.ac.uk/chembl/) databases using the canonical SMILES retrieved from PubChem (https://pubchem.ncbi.nlm.nih.gov/). Liver injury targets (using keywords “Hepatocellular injury”, “Hepatotoxicity”, “Liver diseases”, and “Liver injury”) and offspring developmental toxicity targets (using keywords “Prenatal Exposure”, “offspring female”, “offspring”, and “Developmental toxicity”) were obtained from Genecards (v5.24; https://www.genecards.org/) and CTD databases (2025 update). All targets were standardized using UniProt (release 2025_02; https://www.uniprot.org/). Overlapping targets between BPS, liver injury, and offspring datasets were identified using Venn diagram analysis and considered as potential targets for BPS-induced female offspring liver injury. The intersecting targets were then used to construct a protein–protein interaction (PPI) network via the STRING database (v12.5; https://string-db.org/), which was then visualized and analyzed using Cytoscape (v3.10.4; https://cytoscape.org/), and core targets were identified by calculating topological parameters (Betweenness Centrality, Closeness Centrality, and Degree Centrality) with the cytoNCA plugin. Gene Ontology (GO) and Kyoto Encyclopedia of Genes and Genomes (KEGG) pathway enrichment analyses were performed using the DAVID database (2025 update; https://davidbioinformatics.nih.gov/) to explore the underlying biological mechanisms. Statistical significance was set at *p* < 0.05. Visualization of enrichment results was generated using the Bioinformatics platform (http://www.bioinformatics.com.cn).

### 2.6. Quantitative Real-Time PCR (qRT-PCR)

Total RNA was extracted from rat liver tissue using the AFTSpin Tissue/Cell Fast RNA Extraction Kit for Animal (ABclonal, Wuhan, China) according to the manufacturer’s instructions, including gDNA removal and spin-column purification. Reverse transcription was performed with the ABScript Neo RT Master Mix for qPCR with the gDNA Remover kit (ABclonal, Wuhan, China) under the following conditions: 37 °C for 2 min, 55 °C for 15 min, and 85 °C for 5 min. qRT-PCR was carried out using 2× Universal SYBR Green Fast qPCR Mix (ABclonal, Wuhan, China) on a Bio-Rad CFX96 real-time PCR system (Bio-Rad Laboratories, Inc., Hercules, CA, USA). The 20 μL reaction mixture contained 10 μL of 2× SYBR Green Mix, 2 μL cDNA, 0.4 μL each of forward and reverse primers (10 μM), and 7.2 μL ddH_2_O. The amplification protocol was 95 °C for 10 min, followed by 40–45 cycles of 95 °C for 15 s and 60 °C for 60 s, with melting curve acquisition. The mRNA levels of cholesterol 7-alpha-hydroxylase (Cyp7a1), sterol 27-hydroxylase (Cyp27a1), oxysterol 7-alpha-hydroxylase (Cyp7b1), sterol 12-alpha-hydroxylase (Cyp8b1), 3 beta-hydroxysteroid dehydrogenase type 7 (Hsd3b7), and bile acid-CoA: amino acid N-acyltransferase (Baat) were quantified. Housekeeping gene stability and original Cq values are provided in Table S2. Primer sequences for target genes are listed in Table S3. GAPDH was used as the internal reference gene, and relative gene expression levels were calculated using the 2^−ΔΔCt^ method. The detailed experimental protocol is shown in Text S3.

### 2.7. Western Blotting

Liver tissue was homogenized in ice-cold RIPA lysis buffer (50 mM Tris-HCl, pH 7.4, 150 mM NaCl, 1% NP-40, 0.5% sodium deoxycholate, 0.1% SDS, 1 mM EDTA) supplemented with 1 mM PMSF, 1× protease inhibitor cocktail, and 1× phosphatase inhibitor cocktail, added freshly before use. The homogenate was centrifuged at 12,000× *g* for 15 min at 4 °C, and the supernatant was collected for protein quantification and subsequent Western blot analysis. Protein concentration was quantified using the BCA assay and adjusted to a uniform concentration. Loading buffer was added, and the samples were denatured at 95 °C. SDS-PAGE was performed using a 5% stacking gel and a 10% separation gel, followed by protein transfer onto a polyvinylidene fluoride (PVDF) membrane. The membrane was blocked with 5% bovine serum albumin (BSA) for 2 h and then incubated overnight at 4 °C with primary antibodies (dilution ratios listed in Table S4). Subsequently, the membrane was incubated with a goat anti-rabbit secondary antibody (1:3000 dilution) at room temperature for 2 h. Target protein expression was detected using a standard chemiluminescence reagent, and images were captured with a chemiluminescence imaging system. Band intensity was analyzed using Image Pro Plus software (v7.0; Media Cybernetics, Inc., Bethesda, MD, USA). Standard experimental operations were performed according to Text S4.

### 2.8. Statistical Analysis

Data were analyzed using SPSS v26.0 (IBM Corp., Armonk, NY, USA) and plotted using GraphPad Prism 9.5.1 (GraphPad Software, LLC, Boston, MA, USA). All quantitative data are expressed as mean ± standard error of the mean (SEM). Because each female offspring was selected from a distinct litter, the litter served as the statistical unit for all comparisons. Normality was assessed using the Shapiro–Wilk test, and homogeneity of variances was assessed using Levene’s test. For normally distributed continuous data with homogeneous variances, statistical significance was determined using one-way ANOVA followed by Tukey’s post hoc test; otherwise, the Kruskal–Wallis test followed by Dunn’s post hoc test was applied. For metabolomics data, multiple comparisons were corrected using the Benjamini–Hochberg false discovery rate (FDR) method. Significant metabolite differences were identified based on variable importance in projection (VIP) > 1, raw *p* < 0.05, and FDR-adjusted *q* < 0.05; *q*-values were reported alongside raw *p*-values. Pathway enrichment analysis was performed using the Kyoto Encyclopedia of Genes and Genomes, with significance set at *p* < 0.05.

## 3. Results

### 3.1. Perinatal BPS Exposure Induces Growth Changes in Female Offspring

**Figure 2:**
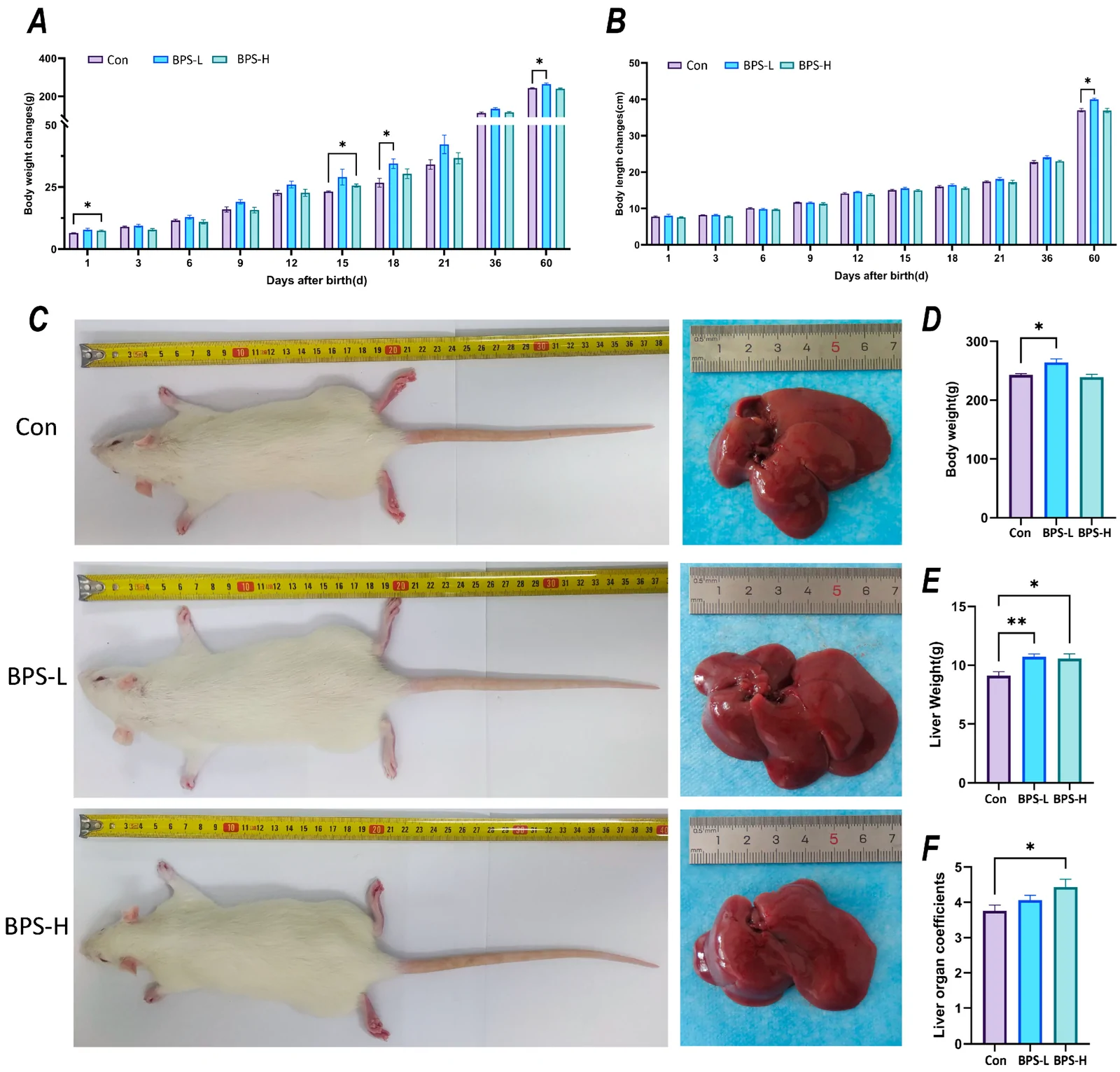
Effects of perinatal exposure to BPS on growth and development, liver weight and appearance of F1 female rats. (**A**) The average body weight change from birth to sexual maturity of F1 female rats produced by each pregnant rat. (**B**) The average body length change from birth to sexual maturity of F1 female rats produced by each pregnant rat. (**C**) The representative appearance and liver macroscopic view of F1 female rats at PND60. (**D**) The body weight of F1 female rats at PND60. (**E**) Liver wet weight of F1 female rats at PND60. (**F**) Liver organ coefficient of F1 female rats at PND60. Data are expressed as mean ± SEM (*n* = 6 litters per group; one female offspring per litter). * *p* < 0.05, ** *p* < 0.01 compare with the control group.

Compared with the Con group, rats in the BPS-L group exhibited significantly increased body weight at PND18 and PND60 (*p* < 0.05) and also showed significantly greater body length at PND60 (*p* < 0.05, Figure 2A,B). Rats in the BPS-H group demonstrated increased body weight at PND1 and PND15 (*p* < 0.05), with no significant difference in body length. By PND60, the BPS-L group exhibited a more pronounced obese phenotype, accompanied by increased macroscopic liver size and weight, although the liver coefficient remained unchanged. In contrast, while the BPS-H group showed higher liver weight and coefficient than the Con group, no significant macroscopic morphological alterations were observed. Litter size and female-to-male ratio at birth were also recorded, and no significant differences were observed among the Control, BPS-L, and BPS-H groups (*p* > 0.05; Table S5).

### 3.2. Perinatal BPS Exposure Induces Hepatotoxicity in Female Offspring

**Figure 3:**
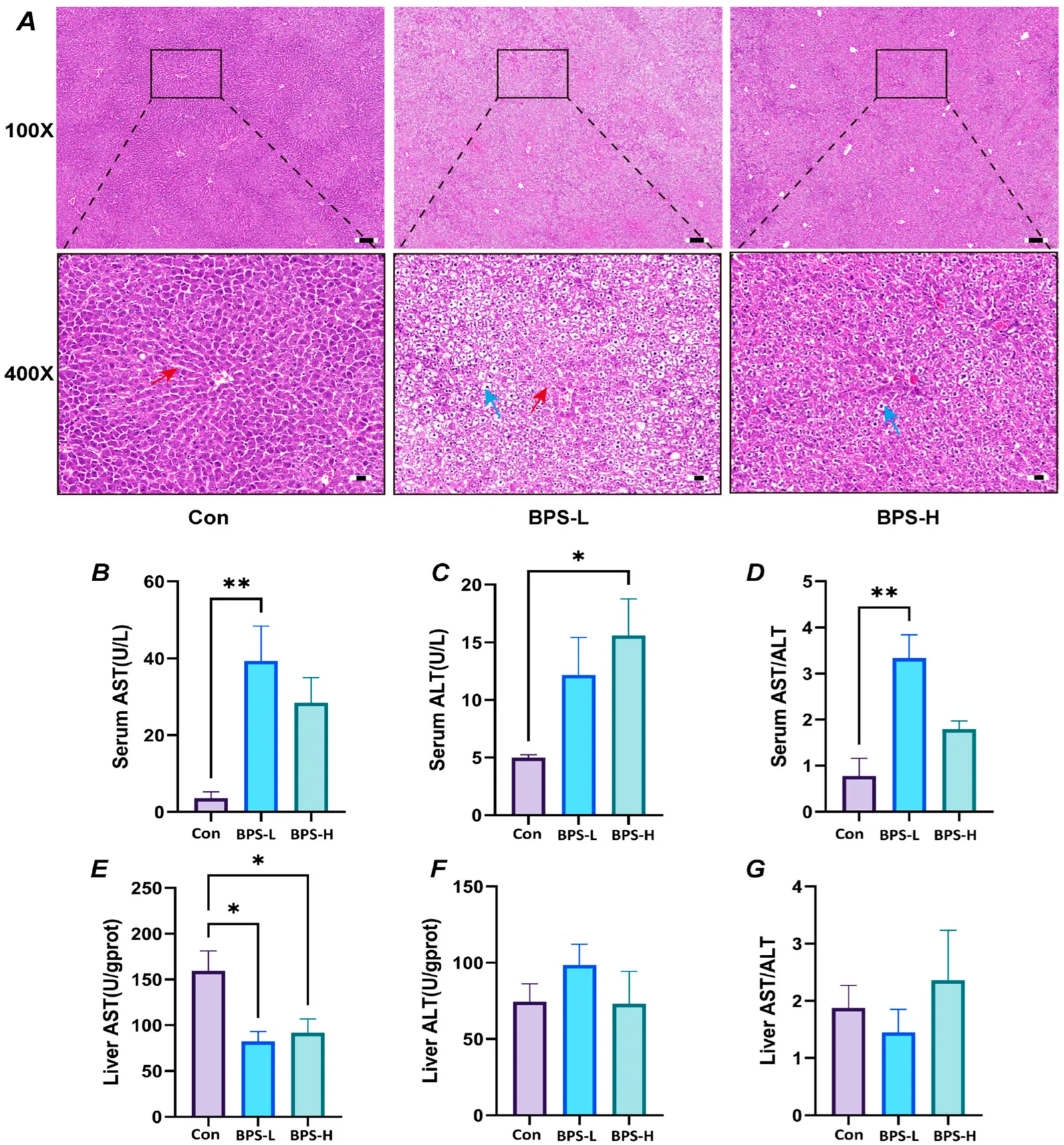
The effect of perinatal exposure to BPS on the morphology of liver tissue and function in F1 female rats. (**A**) Representative hematoxylin and eosin (H&E)-stained liver sections from the Con, BPS-L, and BPS-H groups at PND60 (*n* = 6 litters per group; one female offspring per litter). The upper panels show 100× magnification; the lower panels show 400× magnification of the black-boxed areas. The blue arrow indicates hepatocytes with cytoplasmic vacuolization and displaced nuclei, and the red arrows indicate the hepatic trabeculae and sinusoidal structures. Numerous white, empty-appearing hepatocytes were observed, particularly in the BPS-L group. No obvious inflammatory cell infiltration was observed. Scale bars (inserted black line): 100 μm (100×), 20 μm (400×). (**B**,**E**) Aspartate aminotransferase (AST) levels in serum and liver. (**C**,**F**) Alanine aminotransferase (ALT) levels in serum and liver. (**D**,**G**) AST/ALT levels in serum and liver. Data for AST and ALT are expressed as mean ± SEM (N = 3 litters per group; one female offspring per litter). * *p* < 0.05, ** *p* < 0.01 compare with the control group.

To assess the hepatotoxic effects of perinatal BPS exposure, we first examined liver morphology and function in female offspring at PND60. Liver tissue from the Con group exhibited normal hepatocyte architecture, with hepatocytes arranged radially around the central vein and distinct boundaries between hepatic lobules (Figure 3A). No irregularities were observed in hepatic cords or plates, and the nucleus–cytoplasm separation was intact. In contrast, liver tissue from the BPS-L and BPS-H groups displayed significant alterations in hepatic morphology, with the BPS-L group showing more severe pathological changes. Specifically, the BPS-L group showed almost complete disruption of hepatic lobules, cords, and sinusoids, dissociation of nuclei from the cytoplasm, and hepatocytes of irregular size and shape. Furthermore, darkened, condensed nuclei and cytoplasmic vacuolization were observed. Furthermore, darkened, condensed nuclei and numerous white, empty-appearing hepatocytes were observed, especially in BPS-L; these may indicate lipid accumulation (steatosis) but require Oil Red O confirmation. No obvious inflammatory infiltration was noted.

Consistent with these morphological changes, serum biochemical markers of liver function were significantly altered (Figure 3B–G). In the BPS-L group, compared to the Con group, no significant changes were observed in the serum levels of alanine aminotransferase (ALT), while aspartate aminotransferase (AST) and the AST/ALT ratio were significantly increased (*p* < 0.01). Furthermore, hepatic AST levels in the BPS-L group were significantly decreased (*p* < 0.05) compared to the Con group, whereas no changes were observed in hepatic ALT or hepatic AST/ALT ratio. In the BPS-H group, compared to the Con group, serum ALT levels showed a significant increase (*p* < 0.05), while serum AST and AST/ALT levels remained unchanged. Similarly, compared to the Con group, there was no significant change in hepatic ALT or AST/ALT ratio, but AST levels showed a significant decrease (*p* < 0.05).

### 3.3. Metabolomics Reveals Disruption of Primary Bile Acid Biosynthesis in the Liver of Female Offspring

**Figure 4:**
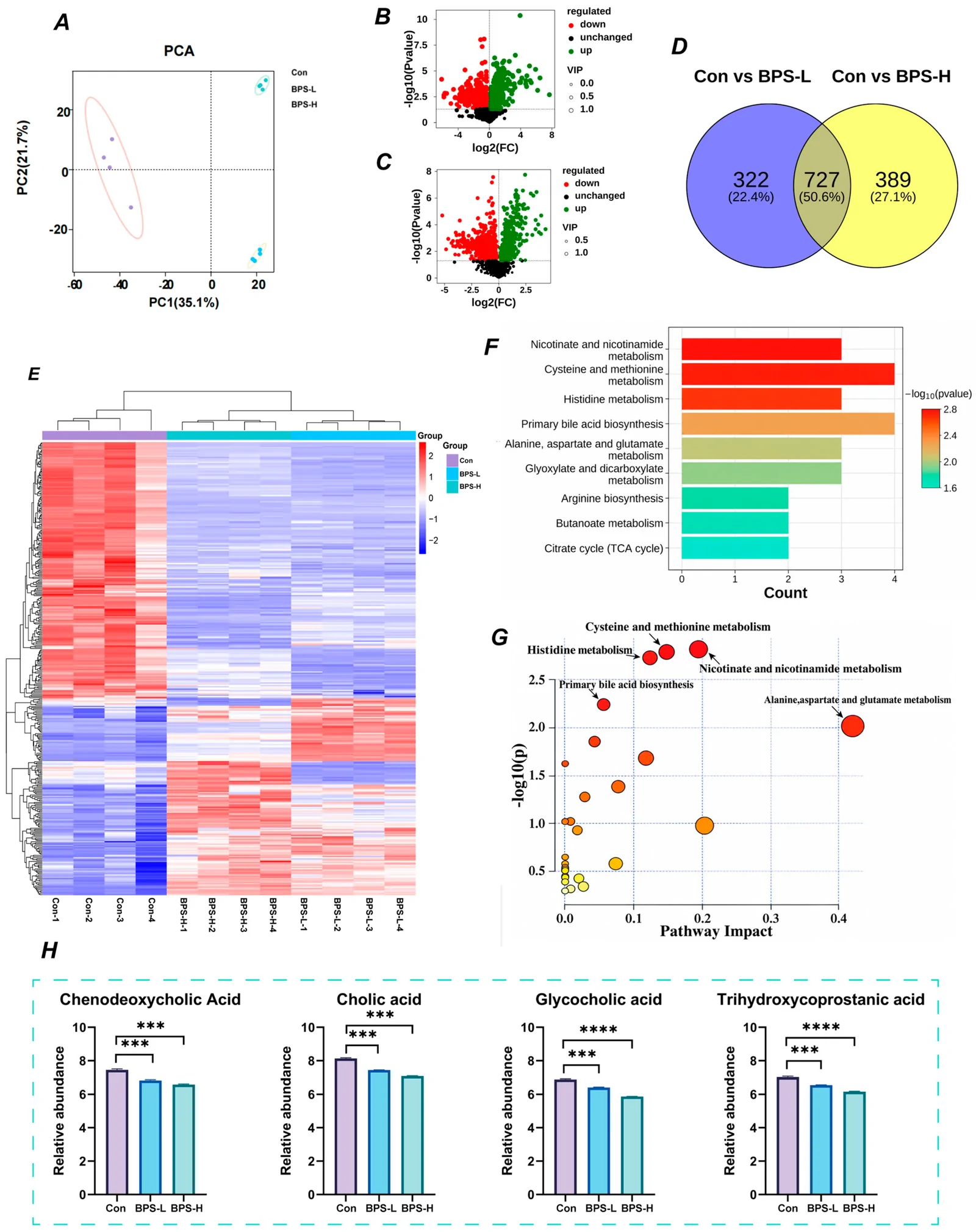
Effects of perinatal exposure to BPS on hepatic off-target metabolomics in F1 female rats. (**A**) Principal component analysis (PCA) plots of each group. (**B**,**C**) Volcano map of “Con vs. BPS-L” and “Con vs. BPS-H”. (**D**) Differential Metabolite Venn plot between “Con vs. BPS-L” and “Con vs. BPS-H”. (**E**) Heat map of differential metabolites. (**F**,**G**) Bar and bubble plots of KEGG enrichment analysis of differential metabolites. The color gradient from yellow to orange to red in panel G indicates increasing statistical significance (i.e., decreasing *p* value), and the size of the circles represents the number of differential metabolites mapped to each pathway. (**H**) Relative abundance of Chenodeoxycholic acid, Cholic acid, Glycoholic acid, and Trihydroxycoprostanic acid. Data are expressed as mean ± SEM (*n* = 4 litters per group; one female offspring per litter). *** *p* < 0.001, **** *p* < 0.0001 compare with the control group.

Principal component analysis revealed distinct clustering and separation patterns among the three groups (Figure 4A), indicating distinct metabolic profiles and the stability within groups. The orthogonal partial least squares discriminant analysis model Q^2^Y values between the Con and BPS-L groups and the Con and BPS-H groups were close to 1 (0.983 and 0.977, respectively), demonstrating high reliability and predictability, as confirmed by 200 permutation tests (Figure S1).

Under the criteria of VIP > 1 and *p* < 0.05, 1049 (481 up- and 568 downregulated) and 1116 (607 up- and 509 downregulated) differential metabolites were identified between the Con and BPS-L groups (Figure 4B) and between the Con and BPS-H groups (Figure 4C). In total, 727 differential metabolites were common across the three groups (Figure 4D). The BPS-L and BPS-H groups shared 368 differential metabolites with similar trends compared to the Con group, with 154 up- and 214 downregulated (Figure 4E). These 368 differential metabolites were subjected to enrichment analysis, which revealed that they were predominantly involved in nicotine and nicotinamide metabolism; cysteine and methionine metabolism; histidine metabolism; primary bile acid biosynthesis; and alanine, aspartate and glutamate metabolism (Figure 4F,G). Four key metabolites involved in primary bile acid biosynthesis were visualized, including chenodeoxycholic acid, cholic acid, glycocholic acid and trihydroxycoprostanic acid (Figure 4H).

### 3.4. Network Toxicology Predicts Cholesterol Metabolism as a Key Pathway of BPS-Induced Liver Injury in Offspring

**Figure 5:**
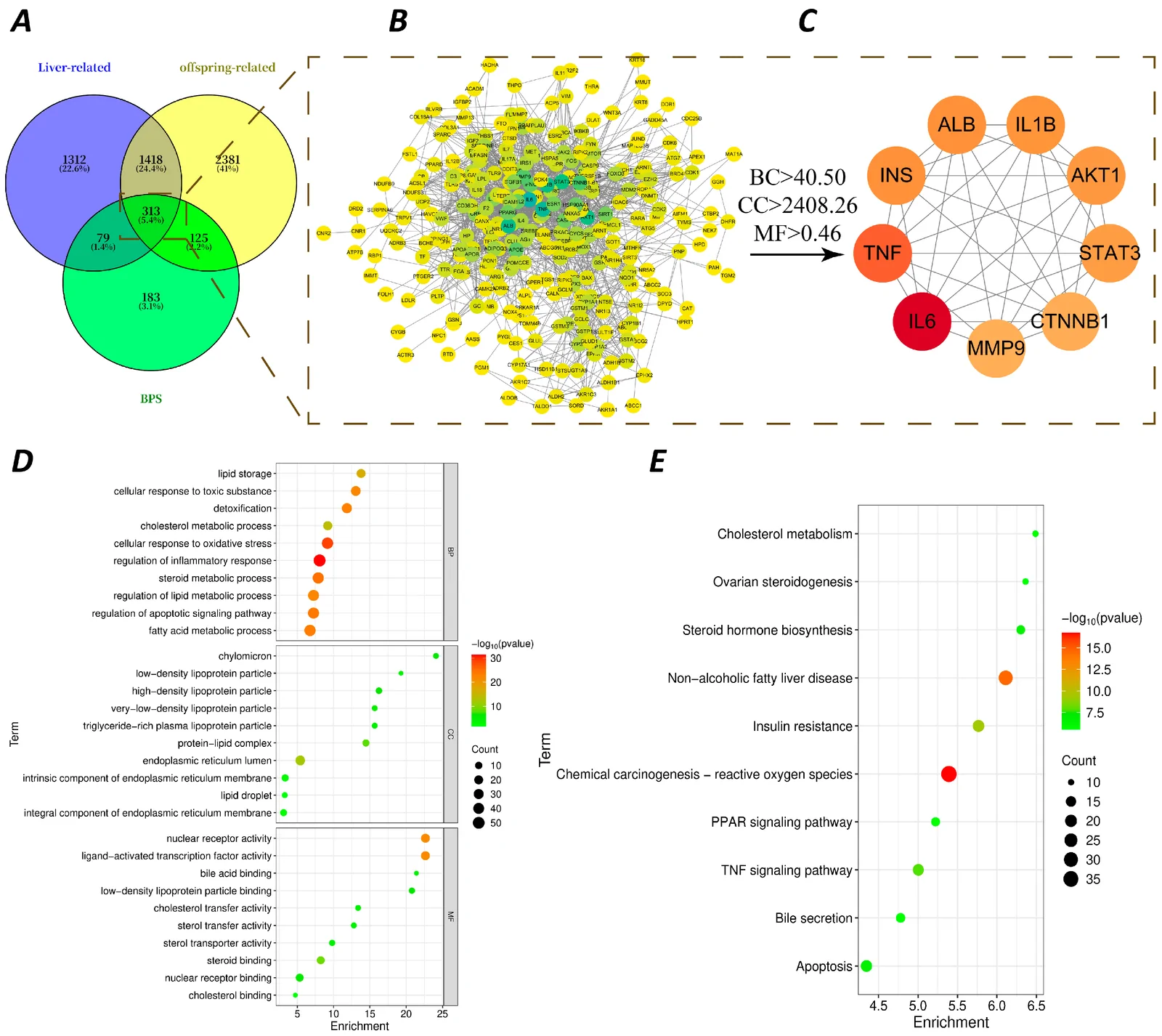
Network toxicology analysis predicts key targets and pathways of BPS-induced liver injury in offspring. (**A**) Venn diagram showing the intersection of BPS targets, liver injury-related targets, and offspring-related targets. A total of 313 overlapping targets were identified. (**B**,**C**) Protein–protein interaction (PPI) network and topology screening. (**D**) GO enrichment analysis of core targets. (**E**) KEGG pathway enrichment analysis of core targets.

To complement the metabolomics findings, network toxicology was employed to predict potential pathways linking BPS exposure to liver injury in offspring. By integrating BPS-related targets (obtained from multiple databases) with liver injury and offspring development targets (Figure 5A), a set of overlapping targets was identified (Figure 5B,C). GO and KEGG enrichment analysis of these targets revealed significant enrichment in cholesterol metabolism, bile secretion, non-alcoholic fatty liver disease, and PPAR signaling pathways (Figure 5D,E). These predictions were consistent with the metabolomics-derived suppression of primary bile acid biosynthesis, further supporting the notion that BPS exposure disrupted cholesterol and bile acid homeostasis in the liver of female offspring.

### 3.5. Validation of Impaired Primary Bile Acid Synthesis Pathway in the Liver of Female Offspring

**Figure 6:**
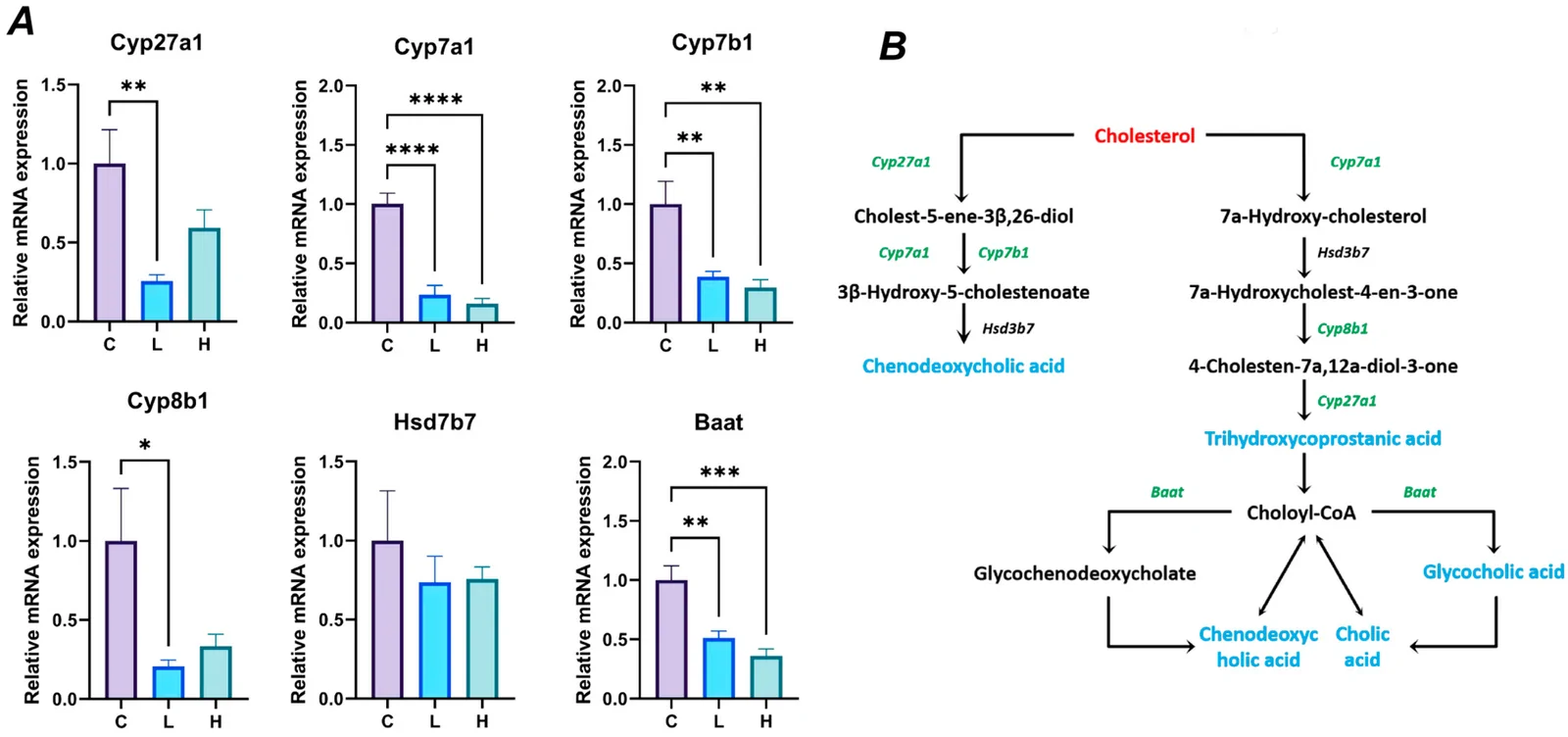
Effects of perinatal exposure to BPS on the mRNA expression of genes involved in the primary bile acid biosynthesis pathway in the liver of F1 generation female rats. (**A**) The relative mRNA levels of Cyp27a1, Cyp7a1, Cyp7b1, Cyp8b1, Hsd3b7, and Baat were quantified by quantitative real-time PCR (qRT-PCR) and normalized to the housekeeping gene GAPDH. (**B**) Schematic representation of the primary bile acid biosynthesis pathway, with the corresponding enzymes indicated in green italics. “C” represents the Con group; “L” represents the BPS-L group; “H” represents the BPS-H group. Data are expressed as mean ± SEM (*n* = 6 litters per group; one female offspring per litter). * *p* < 0.05, ** *p* < 0.01, *** *p* < 0.001, **** *p* < 0.0001 compared with the control group (one-way ANOVA followed by Dunnett’s multiple comparisons test).

Based on the enrichment of primary bile acid biosynthesis in both metabolomics and network toxicology analyses, we further validated key enzymes in this pathway (Figure 6). In comparison with the Con group, the mRNA expression levels of Cyp7a1, Cyp7b1, and Baat were significantly decreased in the BPS-L and BPS-H groups (*p* < 0.01), while no significant change was observed in the mRNA expression level of Hsd3b7. Additionally, the mRNA expression levels of Cyp8b1 and Cyp27a1 were significantly decreased in the BPS-L group (*p* < 0.05), with no change observed in the BPS-H group.

### 3.6. BPS Exposure Disrupts Hepatic Cholesterol Homeostasis by Enhancing Synthesis and Blocking Efflux in Female Offspring

Given the impaired bile acid synthesis observed above, we next examined hepatic cholesterol homeostasis, as cholesterol is the precursor for bile acid synthesis.

#### 3.6.1. Alterations in Serum and Hepatic Lipid Profiles

**Figure 7:**
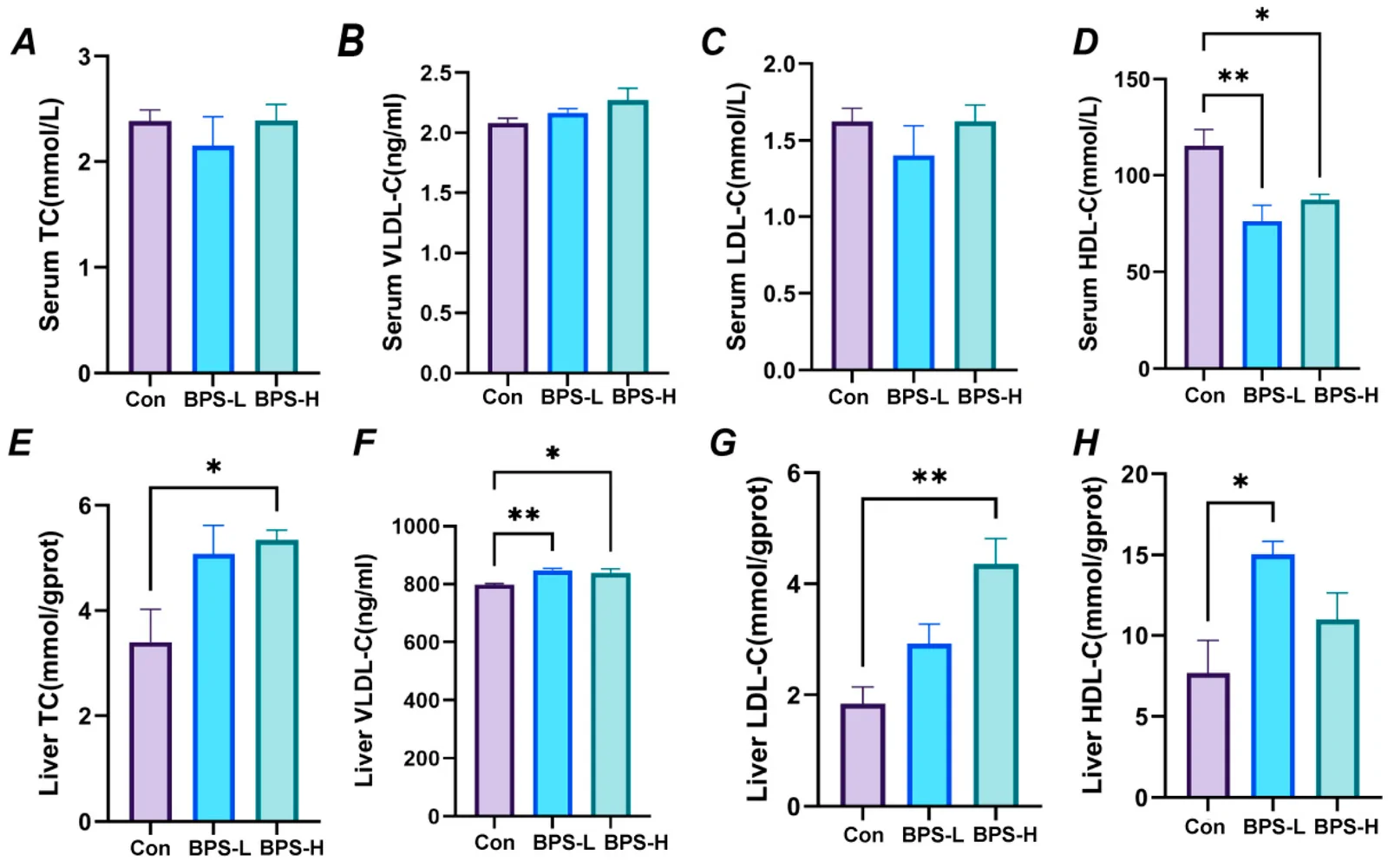
Effects of perinatal exposure to BPS on blood lipids and liver function in F1 female rats. (**A**,**E**) Total cholesterol (TC) levels in serum and liver. (**B**,**F**) Very-low-density lipoprotein (VLDL-C) levels in serum and liver. (**C**,**G**) Low-density lipoprotein (LDL-C) levels in serum and liver. (**D**,**H**) High-density lipoprotein (HDL-C) levels in serum and liver. Data are expressed as mean ± SEM (*n* = 4 litters per group; one female offspring per litter). * *p* < 0.05, ** *p* < 0.01 compare with the control group.

In the BPS-L group, compared with the Con group, there were no significant changes in serum total cholesterol (TC), low-density lipoprotein cholesterol (LDL-C), and very low-density lipoprotein cholesterol (VLDL-C), although a significant decrease in high-density lipoprotein cholesterol (HDL-C) level was observed (*p* < 0.01) (Figure 7A–D). In the liver of the BPS-L group, however, the VLDL-C and HDL-C levels were significantly increased (*p* < 0.05) compared to the Con group, while TC and LDL-C levels remained unchanged (Figure 7E–H). In the BPS-H group, a similar serum HDL-C decrease was observed (*p* < 0.01), with no changes in serum TC, LDL-C, or VLDL-C. Hepatically, TC, VLDL-C, and LDL-C levels were significantly increased (*p* < 0.05), whereas HDL-C showed no significant change.

#### 3.6.2. Dysregulation of Cholesterol Synthesis and Conversion Pathways

**Figure 8:**
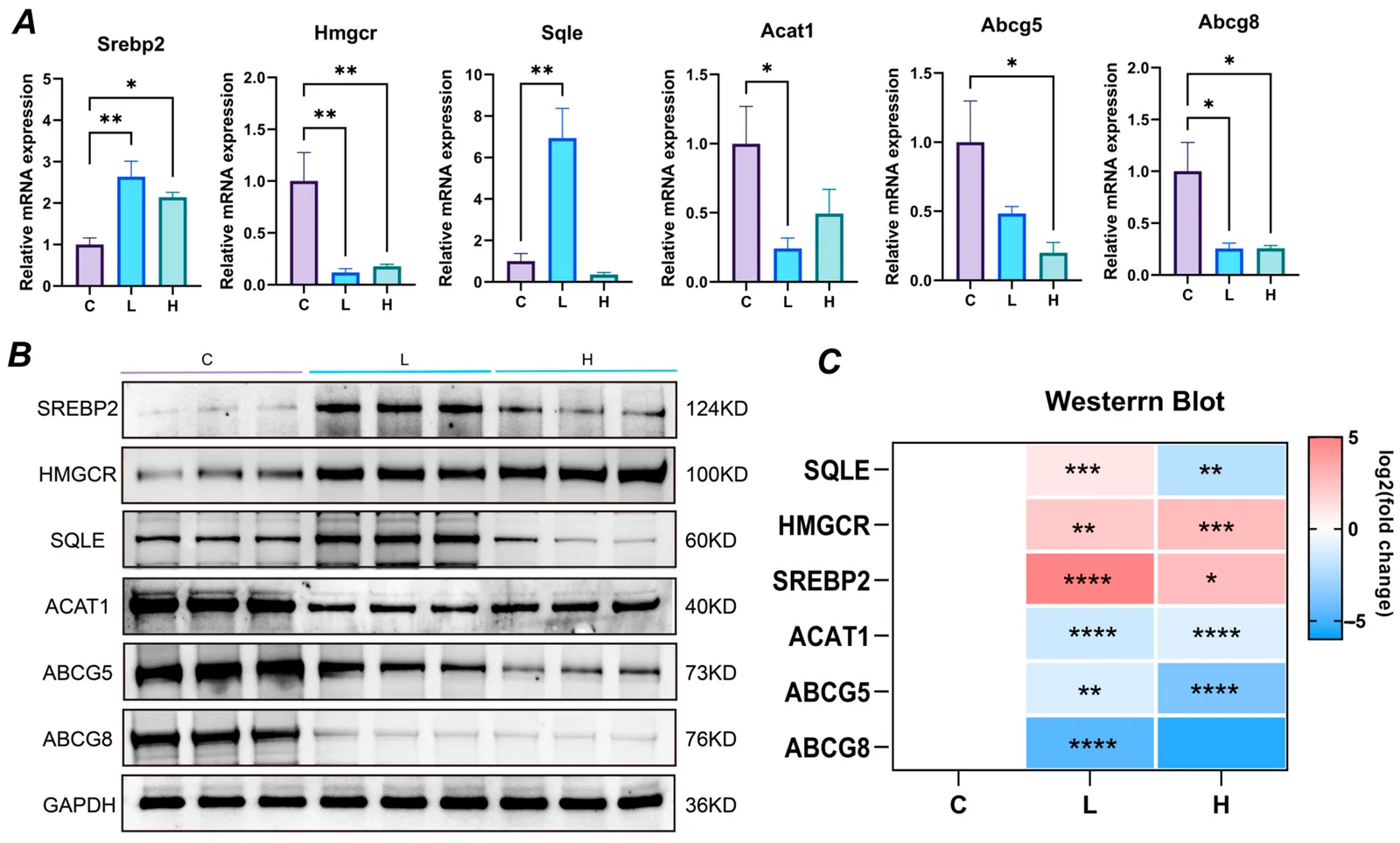
Effects of perinatal exposure to BPS on cholesterol synthesis and transformation in F1 female rats. (**A**) The relative mRNA expression levels of enzymes related to cholesterol synthesis and metabolism quantified by qRT-PCR. Data are expressed as mean ± SEM (*n* = 6 litters per group; one female offspring per litter). (**B**) Western blot images of SREBP2, HMGCR, SQLE, ACAT1, ABCG5, and ABCG8, with GAPDH as the loading control. (**C**) Heatmap showing the log2 (fold change) of protein expression relative to the Con group (set to 0; white). Red indicates upregulation and blue indicates downregulation. Data are expressed as mean ± SEM (*n* = 3 litters per group; one female offspring per litter). “C” represents the Con group; “L” represents the BPS-L group; “H” represents the BPS-H group. * *p* < 0.05, ** *p* < 0.01, *** *p* < 0.001, **** *p* < 0.0001 compare with the control group.

At the transcriptional level (Figure 8A), compared with the Con group, the BPS-L group exhibited increased mRNA expression of sterol regulatory element-binding protein 2 (SREBP2) and squalene epoxidase (SQLE) in the liver of female offspring, along with decreased mRNA expression of 3-hydroxy-3-methylglutaryl-CoA reductase (HMGCR), acetyl-CoA acetyltransferase 1 (ACAT1), and ATP-binding cassette sub-family G member 8 (ABCG8) expression levels showed a decrease, while ABCG5 expression levels remained unchanged (*p* > 0.05). In the BPS-H group, the mRNA expression levels of SREBP2 were increased, whereas HMGCR, ABCG5, and ABCG8 expression levels were decreased; no change was observed in SQLE and ACAT1 expression levels.

Protein expression analysis (Figure 8B,C) largely paralleled these transcriptional changes. In the BPS-L group, SREBP2, HMGCR, and SQLE protein expression levels were significantly increased when compared with the Con group, while ACAT1, ABCG5, and ABCG8 protein expression levels were significantly decreased. SREBP2 and HMGCR protein expression levels in the BPS-H group were significantly increased, while SQLE, ACAT1, ABCG5, and ABCG8 protein expression levels were significantly decreased.

## 4. Discussion

In the present study, we examined the effects of perinatal exposure to environmentally relevant levels of BPS on growth, development, and hepatotoxicity of female offspring. Our findings revealed that perinatal BPS exposure was associated with increased body weight in female offspring at sexual maturity, accompanied by activation of the cholesterol synthesis pathway and inhibition of the cholesterol conversion and efflux pathways in the liver. Specifically, these alterations included upregulation of cholesterol synthesis markers (SREBP2, SQLE, HMGCR) and downregulation of markers related to cholesterol esterification (ACAT1), biliary efflux (ABCG5/ABCG8), and primary bile acid synthesis (Cyp7a1, Cyp27a1). Collectively, these alterations were associated with hepatic cholesterol accumulation and structural and functional liver damage; however, whether cholesterol accumulation is a cause or a consequence of hepatotoxicity remains unclear.

Consistent with previous studies reporting adverse effects of maternal BPS exposure on offspring development [33,34], the present study demonstrated that perinatal BPS exposure induced hepatotoxicity in female offspring. While most existing studies have focused on male offspring [27,35], the effects of BPS on females remain underexplored. Meng et al. [27] found that perinatal BPS exposure significantly increased body weight, liver weight, epididymal white adipose tissue weight, ALT activity, and hepatic TG and total TC levels in male offspring. Another study reported that perinatal BPS exposure in mice altered obesity-related gene expression in adipose tissue of male offspring, including hormone-sensitive lipase, PPARγ, insulin receptor, SOCS3, and adiponectin [35]. Molangiri et al. [36] also reported that gestational BPS exposure induced microvesicular steatosis in male rat offspring, characterized by excess lipid droplets, hepatocyte ballooning, and upregulated ADRP expression, along with increased hepatic IL6, CRP, IL1β, TNFα, and COX2 expression and enhanced lipid peroxidation. In contrast, the present study focused on female offspring and revealed that perinatal BPS exposure induced hepatic cholesterol accumulation and liver injury (e.g., histopathological changes and elevated serum ALT/AST), which were associated with disrupted hepatic cholesterol homeostasis. This study thereby addressed a critical knowledge gap regarding BPS hepatotoxicity in female offspring by defining a novel multi-step cholesterol disruption mechanism, while confirming previously reported effects such as increased body weight and liver injury.

Bisphenol analogues can exert toxic effects on the liver of different species through various mechanisms, including epigenetic modifications and disruption of cellular signaling pathways, gene expression, microbiome and metabolome [28]. To elucidate the core mechanism of liver toxicity induced by BPS in female rat offspring, we employed an integrated approach combining untargeted metabolomics and network toxicology. Untargeted metabolomics analysis revealed significant enrichment of differential metabolites in primary bile acid biosynthesis and amino acid metabolism pathways. Independently, network toxicology predicted 313 potential targets of BPS associated with liver injury and offspring development, with KEGG enrichment identifying cholesterol metabolism, bile secretion, and PPAR signaling as significantly overrepresented pathways. The striking convergence between metabolomics findings (bile acid biosynthesis) and network toxicology predictions (cholesterol metabolism) provided a strong rationale for targeted investigation of cholesterol and bile acid homeostasis. Importantly, although network toxicology also identified inflammatory targets (e.g., TNF, IL6), these were not prioritized because the metabolomics–network convergence pointed more directly to a coordinated disruption of cholesterol synthesis, esterification, and efflux pathways, a mechanistic axis that was subsequently validated by qPCR and Western blot analyses.

Primary bile acids, as important metabolic products of cholesterol in the liver, are synthesized through the regulation of key enzymes such as Cyp7a1 and Cyp27a1 [37]. Cyp7a1 is the rate-limiting enzyme in the classical bile acid synthesis pathway, and its activity directly determines the efficiency of cholesterol conversion to bile acids, while Cyp27a1 plays a core role in the alternative pathway and both cooperate to maintain the balance between bile acid synthesis and cholesterol metabolism [38,39]. Mao et al. [40] reported that low-dose BPS exposure (50 μg/kg/day for 28 days) altered bile acid profiles in adolescent male SD rats, with decreased taurochenodeoxycholic acid and deoxycholic acid, suggesting impaired bile acid synthesis and enterohepatic recycling. The results of the present study showed that the mRNA expressions of Cyp7a1, Cyp7b1 and Baat in the livers of female offspring exposed to BPS-L and BPS-H were significantly downregulated. In the low-dose group, the expressions of Cyp8b1 and Cyp27a1 were also significantly reduced. This finding is consistent with the conclusion of Evangelakos et al. [41] who reported that inhibition of bile acid synthase leads to sluggish cholesterol metabolism and insufficient bile acid accumulation. Suppression of the bile acid synthesis pathway is known to trigger feedback activation of cholesterol synthesis and accumulation of unconverted cholesterol in the liver, while simultaneously compromising the hepatoprotective functions of bile acids [42]. Such disruption can trigger oxidative stress and mitochondrial damage, ultimately contributing to liver inflammation and fibrosis [42]. The pathological changes observed in our study, including hepatic lobule disintegration, cytoplasmic vacuolation, and nuclear shrinkage, further support the notion that BPS disrupted cholesterol-bile acid homeostasis by inhibiting primary bile acid synthesis, which would be associated with liver damage. In addition to the biochemical changes, histological examination revealed an apparent inconsistency: the BPS-L group showed more severe vacuolization than the BPS-H group, whereas the BPS-H group had higher hepatic total cholesterol and a more yellowish liver appearance. This discrepancy may reflect non-monotonic dose responses [43], differences in the type and distribution of accumulated lipids (e.g., triglycerides vs. cholesterol) [44], or compensatory responses at the higher dose. It is also possible that cytoplasmic vacuolization does not exclusively represent lipid droplets but may also reflect other forms of cellular injury, such as organelle swelling or water accumulation. Consistent with this, Molangiri et al. [36] reported that gestational BPS exposure induced microvesicular steatosis in male rat offspring, characterized by excess lipid droplets and hepatocyte ballooning. However, without Oil Red O staining or electron microscopy, the precise nature of these vacuoles cannot be determined. Future studies incorporating these techniques are needed to clarify the relationship between histological vacuolization and hepatic lipid accumulation.

The synthesis of cholesterol commences with acetyl-CoA, which is catalyzed by HMG-CoA reductase to form mevalonic acid. This subsequently undergoes multiple reactions involving SQLE to ultimately yield cholesterol [45]. During the conversion stage, ACAT1 esterifies free cholesterol into cholesterol esters, reducing the toxicity of free cholesterol in the cells and providing raw materials for the assembly of VLDL-C [46]. For excretion, the ATP-binding cassette transporter G subfamily 5/8 (ABCG5/ABCG8) complex transports excess cholesterol from the liver to bile, or secretes it into the peripheral blood [47]. The results of this study indicate that exposure to BPS interfered with cholesterol metabolism in the livers of female offspring, manifested as altered expression of genes involved in cholesterol synthesis, conversion, and excretion. At the protein level, the BPS-L group exhibited significantly higher expression of SREBP2, HMGCR, and SQLE, while ACAT1, ABCG5 and ABCG8 showed significantly lower expression; in the BPS-H group, SREBP2 and HMGCR were significantly upregulated, while SQLE, ACAT1, ABCG5 and ABCG8 were downregulated. This finding was consistent with previous studies demonstrating that direct BPS exposure in adult mice disrupts hepatic cholesterol metabolism, leading to dysregulation of lipid synthesis-related proteins [48]. Li et al. [44] demonstrated that low-dose BPS (10 mg/kg/day) upregulated SREBP1 and promoted triglyceride synthesis in male SD rats, whereas high-dose BPS (50 mg/kg/day) induced cholesterol accumulation despite inhibiting the mRNA expression of Srebp2, Hmgr, and Hmgs, suggesting that different doses of BPS activate different SREBP isoforms and lead to distinct lipid accumulation patterns. The observed upregulation of cholesterol synthesis genes (SREBP2, HMGCR) aligns with a well-established mechanism by which imbalances in cholesterol metabolism can lead to hepatic cholesterol accumulation [49]. This convergence of evidence across different compounds and generations suggests that interference with cholesterol homeostasis may represent a common pathway through which diverse environmental contaminants induce hepatotoxicity, further supporting that the multi-step disruption of cholesterol metabolism by BPS contributes to cholesterol accumulation in the liver of female offspring.

Cholesterol metabolism disorder can lead to secondary abnormalities in liver function markers (ALT, AST) and lipoprotein metabolism by damaging the structural integrity of liver cells and interfering with substance transport functions. Moreover, under low and high doses of BPS exposure, this secondary effect shows significant dose-specific differences. The differences in serum AST and ALT may reflect the location and degree of liver cell damage [50]. ALT is mainly located in the cytoplasm of liver cells, while AST is mainly present in the mitochondrial matrix. When liver cells are damaged, enzymes in the cytoplasm or mitochondrial matrix will be released into the blood, causing changes in serum enzyme activity. Conversely, the enzyme activity in the liver is reduced due to enzyme release or blocked synthesis [51]. In this study, the serum AST and AST/ALT ratio of female offspring in the low-dose BPS exposure group were significantly increased, and liver AST was significantly decreased. In the high-dose group, only serum ALT was significantly increased, while liver AST remained significantly decreased. This difference may be related to the toxic effect of cholesterol accumulation. At low doses, the initial accumulation of cholesterol in the liver mainly damaged mitochondria, leading to the release of AST into the blood and consequently increased serum AST; while at high doses, excessive accumulation of cholesterol further damaged the integrity of the plasma membrane, causing substantial ALT release from the cytoplasm, thus resulting in a significant increase in serum ALT. Meanwhile, mitochondrial damage persists, keeping liver AST at a low level. This damage pattern may be associated with the aggravation of cholesterol accumulation with increasing dose. However, AST is not liver-specific and can also originate from skeletal muscle and heart, where its activity can exceed that in the liver [52,53]. Thus, the elevated serum AST cannot be attributed solely to hepatocyte injury. Nevertheless, the concurrent changes in ALT, hepatic AST/ALT ratio, histopathological findings, and liver lipid data collectively support the interpretation that the observed alterations primarily reflect hepatic injury associated with BPS exposure. Future studies should include creatine kinase (CK) and lactate dehydrogenase (LDH) to clarify the tissue source.

Furthermore, lipoproteins serve as the core carriers for the transport of cholesterol within the body, and their level changes are directly regulated by the cholesterol metabolism of the liver and the secretion functions of cells [54]. In the present study, at low doses of BPS, cholesterol synthesis markers were slightly upregulated and ACAT1 expression was downregulated, which was associated with an increase in free cholesterol in the liver. Although this can stimulate the assembly of VLDL-C, the insufficient expression of ACAT1 has been shown to prevent the maturation of VLDL-C, which may explain its accumulation in the liver observed in this study [55]. At the same time, the observed transient increase in HDL-C in the liver was likely due to the increased demand for reverse cholesterol transport [56]. However, the subsequent decrease in serum HDL-C likely reflects inhibition of hepatocyte synthesis function [57]. At high doses of BPS, cholesterol synthesis was further intensified and the expression of ABCG5 and ABCG8 was significantly decreased, which was associated with marked accumulation of TC in the liver. Under these conditions, VLDL-C accumulated both due to the excess of synthesis raw materials and secretion disorders, while LDL-C may also have increased as a result of accumulation of VLDL-C metabolic intermediates. Meanwhile, serum HDL-C remained at a low level due to decreased liver synthesis capacity.

Furthermore, we observed that the damage caused by BPS exposure did not exhibit a typical dose–response relationship, with low-dose exposure often inducing more pronounced changes than high-dose exposure. This non-monotonic dose response is a hallmark of endocrine-disrupting chemicals [47] and may reflect the complexity of BPS’s biological actions. At 0.05 mg/kg, BPS may effectively disrupt endocrine homeostasis without triggering overt cytotoxicity, leading to maximal metabolic perturbation. In contrast, at 20 mg/kg, more extensive cellular stress may activate compensatory mechanisms, such as detoxification pathways, autophagy, or survival-oriented metabolic reprogramming, that partially offset the toxic effects. Alternatively, high-dose exposure may directly compromise cell membrane integrity, as suggested by elevated serum ALT, which could overshadow more subtle receptor-mediated effects. These possibilities require further investigation, including studies with additional dose levels and internal exposure measurements, to clarify the dose–response relationship and the mechanisms underlying BPS-induced hepatotoxicity.

Nevertheless, several limitations of this study warrant comment. First, internal BPS exposure levels in maternal or offspring tissues were not measured, which limits precise dose–response assessment and extrapolation to exposure scenarios. Second, although BPS is known to exert endocrine-disrupting effects [20,21], hormone levels were not quantified in F0 dams and F1 offspring. Wang et al. [58] showed that perinatal exposure to a BPS derivative 4-hydroxyphenyl 4-isopropoxyphenylsulfone (BPSIP; 5 μg/kg/day) disrupted hepatic cholesterol metabolism sex-specifically in ICR mice, with higher liver cholesterol in females and lower in males, and that ERα binding to Hmgcr, Pcsk9, and Abcg5 was attenuated in females but enhanced in males. Given that estrogen signaling plays a critical role in regulating hepatic cholesterol metabolism [59], the absence of hormonal data precludes direct linkage between endocrine disruption and the observed cholesterol dyshomeostasis. Thus, future studies should include hormone profiling in both F0 dams and F1 offspring. Third, no male offspring from the same experiment were available, so a direct male and female comparison under identical experimental conditions could not be performed; comparisons with published male data are therefore indirect and should be interpreted cautiously. Fourth, the relationship between the doses used in this study and actual human exposure remains unclear. Species differences in BPS metabolism, exposure routes, bile acid pool composition, and dose conversion should be considered, and BPS currently has no official reference dose (RfD). Future studies could incorporate complementary assays, including bile acid profiling, cholesterol flux/tracer studies, Oil Red O staining, hepatic triglyceride quantification, liver function tests (alkaline phosphatase, bilirubin, and indocyanine green clearance), and direct male–female comparisons, to further elucidate the mechanisms of BPS-induced hepatotoxicity. Addressing these limitations in future research will deepen our understanding of BPS hepatotoxicity and its implications for human health.

In summary, we propose that perinatal BPS exposure induces cholesterol accumulation and hepatotoxicity in female offspring through a dual mechanism involving enhanced cholesterol synthesis via upregulation of SREBP2/HMGCR/SQLE, and suppressed cholesterol clearance via inhibition of cholesterol esterification (ACAT1), biliary efflux (ABCG5/ABCG8), and bile acid synthesis (Cyp7a1/Cyp27a1). The low dose (0.05 mg/kg/day) was based on the U.S. EPA RfD for BPA, since BPS lacks an official RfD. Due to species differences in exposure, metabolism, bile acid composition, and dose conversion, human extrapolation should be cautious; these findings may aid BPS risk assessment but need validation in human studies. These findings provide novel insights into the hepatotoxic effects of BPS on female offspring and highlight the need for further investigation into the dose–response relationships, direct male–female comparisons, and the hormonal and sex-specific mechanisms underlying BPS-induced hepatotoxicity.

## 5. Conclusions

Our study results demonstrate that perinatal exposure to BPS is associated with hepatotoxicity in female rat offspring, accompanied by altered expression of genes involved in hepatic cholesterol synthesis and conversion, as well as cholesterol accumulation and liver injury. However, the causal relationship between cholesterol accumulation and liver injury requires further investigation. Because BPS currently has no official RfD and species differences may limit direct human extrapolation, these findings should be interpreted as potential implications rather than direct evidence of human risk. Given the widespread use of BPS and its potential for environmental exposure, further studies are warranted to assess its broader impacts on ecosystems and human health.

## Data Availability

The data presented in this study are available on request from the corresponding authors.

## Supplementary Materials

The following supporting information can be downloaded at https://www.mdpi.com/article/10.3390/toxics14100878/s1, Text S1: HE staining procedure; Text S2: Non-target Metabolomics; Text S3: RT-qPCR experiment procedure; Text S4: Western Blot; Figure S1: Effects of perinatal exposure to BPS on hepatic off-target metabolomics in F1 female rats; Table S1: The main kits and antibodies used in the present study; Table S2-1: Housekeeping gene stability; Table S2-2: Original Cq value of housekeeping genes; Table S3: Information for primer-pairs used in SYBR Green-based quantitative real-time PCR assays; Table S4: Antibody dilution ratio; Table S5: Litter size and female-to-male ratio of F1 offspring.
