# Supplementary material for: Perinatal Bisphenol S Exposure Induces Hepatotoxicity in Female Rat Offspring: Insights into Disrupted Hepatic Cholesterol Homeostasis
Source: Toxics. 2026 Sep 30;14(10):878. doi: 10.3390/toxics14100878 (PMC13709647; doi:10.3390/toxics14100878)
Supplement: Supplementary file 1 [file toxics-14-00878-s001.zip › toxics-4535298-supplementary.pdf]

Supplementary materials

Perinatal Bisphenol S Exposure Induces Hepatotoxicity in Female Rat Offspring: Insights into Disrupted Hepatic Cholesterol Homeostasis

Hongming Huang [a,1], Jingyi Zhang [b,1], Jing Jin [c,d,e,1], Ying Xie [a], Rui Pan [b], Min Zhao [a,c], Biran Zhu [a,c]* and Jianghuan Hua [a,c]*

[a]School of Basic Medical Sciences, Hubei University of Chinese Medicine, Wuhan 430065, China

[b]Department of Pediatrics, Institute of Pediatric Diseases and Rare Diseases, Xiangyang Central Hospital, Affiliated Hospital of Hubei University of Arts and Science, Xiangyang 441021, China

[c]Hubei Shizhen Laboratory, Wuhan 430061, China

[d]The First Clinical Medical School，Hubei University of Chinese Medicine, Wuhan 430065, China

[e]Hubei Provincial Hospital of Traditional Chinese Medicine, Wuhan 430061, China

[1] These authors contributed equally to this work.

*Correspondence: Corresponding author: **Jianghuan Hua** - School of Basic Medical Sciences, Hubei University of Chinese Medicine, Wuhan 430065, China; Hubei Shizhen Laboratory, Wuhan 430061, China. Email: jhua@hbucm.edu.cn. **Biran Zhu** - School of Basic Medical Sciences, Hubei University of Chinese Medicine, Wuhan 430065, China; Hubei Shizhen Laboratory, Wuhan 430061, China. Email: zhubir@hotmail.com

Number of pages: 15; number of Texts: 4; number of Figures: 1; number of Tables: 5.

Text S1: HE staining procedure

1. Tissue dehydration: Tissue samples were subjected to a series of gradient alcohol dehydration treatments, including immersion in 75% alcohol for 4 hours, followed by 85% alcohol (2 hours), 90% alcohol (1.5 hours), 95% alcohol (1 hour), and then through two rounds of absolute ethanol, each for 0.5 hours.

2. Tissue transparency: After dehydration, the tissue blocks needed to be made transparent. In this step, a clearing agent (such as xylene) could be mixed with both dehydrants and paraffin so that paraffin permeation into the tissue was facilitated. Specifically, the tissue was immersed in a 1:1 mixture of absolute ethanol and xylene for 10 minutes, followed by two rounds of pure xylene, each for 10 minutes.

3. Dipping wax: The cleared tissue blocks are then sequentially placed in three baths of 60°C paraffin for wax infiltration, each bath for 1 hour, so that complete paraffin penetration was ensured.

4. Embedding: The wax-infiltrated tissue blocks were enclosed in solid paraffin blocks. The temperature of the paraffin used for embedding needed to be set slightly higher than that used for wax infiltration to ensure complete fusion between the tissue blocks and paraffin blocks.

5. Slicing and baking: The embedded paraffin blocks were sliced using a Leica pathological microtome, and the slices were then placed in a 40°C water bath to be flattened. The slices were accurately adhered to an anti-slip slide by tilting and inserting the slide. Subsequently, the slides were placed in a 60°C oven for 3 hours so that the sections were fixed.

6. Slices deparaffinisation: The slides were subjected to a series of dewaxing steps, including immersion in xylene I, xylene II, and xylene III for 20 minutes each, followed by a series of absolute ethanol and gradient alcohol treatments, and finally were rinsed in distilled water. The slides were then stained with hematoxylin for 5 minutes, rinsed in tap water until blue, stained with eosin for 5 minutes, and rinsed quickly in tap water for 30 seconds.

7. Gradient dehydration and microscopic observation: After staining, the slides were subjected to another gradient dehydration process, including immersion in 95%

alcohol for 30 seconds and 1 minute, followed by two rounds of absolute ethanol for 5 minutes each, and finally were subjected to clearing treatment in xylene I and xylene II for 5 minutes each. After natural drying, the slides were coverslipped with neutral gum, and finally observed under a microscope.

Text S2: Non-target Metabolomics

1. Experimental reagents and instruments

List of experimental reagents

Name	CAS	Purity	Brand
Methanol	67-56-1	LC-MS Grade	Merck
Acetonitrile	75-05-8	LC-MS Grade	Merck
L-2-Chloro-L-phenylalanine	103616-89-3	≥98%	Shanghai Aladdin
Formic Acid	64-18-6	LC-MS Grade	TCI

List of experimental instruments

Instrument	Model	Brand
Chromatograph	Ultim3000	Thermo
Mass Spectrometer	Orbitrap Exploris 480	Thermo
Chromatographic Column	Acquity UPLC HSS T3 1.8um 2.1*100mm	Waters

2. Metabolite extraction

An appropriate volume of extraction solvent and magnetic beads were added for grinding and sonication. After static centrifugation, the supernatant was collected for vacuum drying. Subsequently, 160 μL of extraction solvent (acetonitrile-water ratio: 1:1) was added for resuspension, followed by detection using the instrument.

3. Instrument detection

The samples were detected on a Thermo Ultim3000 High-Performance Liquid Chromatograph coupled with a Thermo Orbitrap Exploris 480 High-Resolution Mass

Spectrometer (LC-MS). Chromatographic separation was performed on a Waters Acquity UPLC HSS T3 column (1.8 μm, 2.1 × 100 mm), and the injection volume was 1 μL. For positive ion mode (POS), mobile phase A was 0.1% formic acid in water, and mobile phase B was 0.1% formic acid in acetonitrile. For negative ion mode (NEG), mobile phase A was 0.1% formic acid in water, and mobile phase B was 0.1% formic acid in acetonitrile. The mobile phase gradient was programmed at a flow rate of 400 μL/min as follows: 98% A and 2% B at 0.0 min, maintained until 0.25 min; 2% A and 98% B at 10.0 min, maintained until 13.0 min; 98% A and 2% B at 13.1 min, maintained until 15.0 min. The scan range was set to m/z 67–1000. The ESI source settings were as follows: the spray voltage was 3.5 kV for positive ion mode and 2.5 kV for negative ion mode; the sheath gas flow was 50 arb; the auxiliary gas flow was 10 arb; the sweep gas flow was 1 arb; the ion transfer tube temperature was 325°C; and the vaporizer temperature was 350°C. The polarity was set to positive and negative. MS/MS data-dependent acquisition was used for secondary scanning.

4. Metabolite qualitative and quantitative analysis

The raw data (.raw) files were imported into the CD library search software for preliminary screening of retention time, mass-to-charge ratio, and other parameters. Peak alignment across samples was performed using a retention time deviation of 0.2 min and a mass deviation of 5 ppm to improve identification accuracy. Peak extraction was performed using a mass deviation of 5 ppm, a signal intensity deviation of 30%, a signal-to-noise ratio of 3, a minimum signal intensity of 100,000, and ion summation. Peak areas were quantified, and target ions were integrated. Molecular formulas were predicted based on molecular ion peaks and fragment ions, and the predicted formulas were then compared with those in the mzCloud, MZvault, and ChemSpider databases to obtain the final identification and quantification results.

5. Differential metabolite screening and pathway enrichment

Differential metabolites were screened based on VIP > 1, |log2FC| > 1, and $p < 0.05$. KEGG pathway enrichment analysis was performed on the differential metabolites using the BMKCloud platform, and pathways with $p < 0.05$ were considered significantly enriched. If FDR correction was applied, pathways with FDR < 0.05 were

considered significantly enriched.

Text S3: RT-qPCR experiment procedure

1. RNA extraction

1.1. Fresh tissue (20 mg) was added to 500 μL of Buffer RL1 and then homogenised using a glass homogeniser or an electric homogeniser until there were no visible tissue clumps.

1.2. The lysed sample was transferred to a gDNA clean-up column with a collection tube in place, centrifuged at 13,000 rpm for 1 min, the gDNA clean-up column was discarded, and the filtrate was collected.

1.3. Anhydrous ethanol (0.5 times the volume of the filtrate) was added, and then the mixture was immediately blown and mixed.

1.4. The above mixture was added to an RNase-free adsorption column with a collection tube in place, centrifuged at 13,000 rpm for 30 s, and the filtrate was poured off.

1.5. The RNase-free column was put back into the collection tube, 700 μL of Protein Removal Solution PR2 was added to the column, centrifuged at 13,000 rpm for 30 s, and the filtrate was poured off.

1.6. The RNase-free adsorbent column was placed back into the collection tube, 500 μL of rinse solution WB2 was added to the adsorbent column (after it was ensured that anhydrous ethanol had been added before use), centrifuged at 13,000 rpm for 30 s, and the filtrate was poured off.

1.7. The RNase-free adsorbent column was placed back into the collection tube and the empty tube was centrifuged at 13,000 rpm for 2 min so that the rinse solution WB2 remaining in the adsorbent column was removed (the empty tube could be left in a fume hood for 5 min until the ethanol had completely evaporated).

1.8. The RNase-free adsorbent column was removed and put into a 1.5 mL RNase-free centrifuge tube, 30-100 μL of RNase-free dd H_2O was added to the middle of the adsorbent column, and then the column was centrifuged at 13,000 rpm

for 1 min so that the RNA was eluted out after it had been left at room temperature for 2 min.

1.9. Extracted RNA could be used directly in downstream experiments or could be stored at -80°C.

2. mRNA reverse transcription to cDNA

2.1 Reverse transcription reaction system:

Components	added
4X ABScript Neo RT Master Mix	5 μL
20X gDNA Remover Mix	1 μL
Total RNA	10 pg-1μg
RNase-free ddH2O	Up to 20

2.2 Reaction conditions:

Temperature	Time
37℃	2 min
55℃	15 min
85℃	5 min
4℃	hold

3. Real-time fluorescent quantitative PCR assay

3.1 Reaction system:

Components	Added volume
2X Universal SYBR Green Fast qPCR Mix	10 μL
cDNA	2 μL
Forward primer (10 μM)	0.4 μL
Reverse primer (10 μM)	0.4 μL
ddH_2O	7.2 μL

3.2 Reaction procedures:

Items	Temperature	Time	Cycle number
premutability	95℃	10 min	1

denaturation	95℃	15 s	40-45
Annealing extension	60℃	60 s	
	95℃	15 s	
Melting curve acquisition	60℃	60 s	1
	95℃	15 s	

Text S4: Western Blot

1. Preparation of protein samples

1.1. Extraction of total protein from liver tissue

A small piece of freshly cut liver tissue (20 mg) was placed into a pre-cooled 2 mL microcentrifuge tube containing clean stainless steel beads. Ice-cold RIPA lysis buffer (50 mM Tris-HCl, pH 7.4, 150 mM NaCl, 1% NP-40, 0.5% sodium deoxycholate, 0.1% SDS, 1 mM EDTA) was freshly supplemented with 1 mM PMSF, 1× protease inhibitor cocktail, and 1× phosphatase inhibitor cocktail. Then, 200 μL of the supplemented lysis buffer was added to each tube. The mixture was homogenized using an automated homogenizer. After homogenization, the tubes were kept on ice for 30 minutes to ensure complete lysis. The homogenate was then transferred to a 1.5 mL centrifuge tube and centrifuged at 12,000 × g for 15 min at 4°C. The supernatant was carefully collected, and protein concentration was determined using a BCA assay. The remaining supernatant was aliquoted into 0.5 mL centrifuge tubes and stored at -80°C for further analysis.

1.2. Determination of protein concentration

The protein samples were appropriately diluted (1 μL of each sample was mixed with 19 μL of PBS, resulting in a 20-fold dilution). Concurrently, a series of BSA standards were prepared at concentrations of 1, 0.8, 0.6, 0.4, and 0.2 mg/ml. Both the diluted standards and samples were aliquoted into a 96-well plate, with two replicate wells for each standard and three replicate wells for each sample, containing 20 μL per well. Two additional wells containing only PBS were used as blank controls. The BCA kit was utilized according to the manufacturer's instructions. Reagents A and B were mixed at

a ratio of 50:1, and 200 μL of this mixture was added to each well. After incubation at 37°C for 30 minutes in the dark, the OD_{562} values were measured using a DG-3022A microplate reader. A straight-line regression equation was constructed based on the standard protein concentrations and their corresponding OD values, which was then used to calculate the protein concentrations of the samples.

1.3. protein denaturation

The extracted protein supernatant was mixed with 5 × protein loading buffer in a 4:1 volume ratio and heated in boiling water for 10 minutes. The transparency and viscosity of the samples were checked; if not transparent or sticky, the boiling time was extended. The denatured samples were then cooled to room temperature and stored at -20°C.

2. Electrophoresis: The prepared gel was mounted onto the electrophoresis tank, and the electrophoresis buffer was poured into the reservoir. With a micropipette, protein samples and the marker were loaded into the sample wells, with a total protein amount of 10 μg per well. The electrophoresis was initially performed at 80 V for 30 minutes through the stacking gel, followed by 120 V for 1 hour through the resolving gel. The duration could be adjusted based on the position of the target bands.

3. Transfer: The gel was carefully removed, and a PVDF membrane was cut to match the gel dimensions. The membrane was soaked in methanol for 30 seconds and then transferred to the transfer buffer for equilibration. The transfer sandwich was assembled in the following order: sponge, filter paper, equilibrated PVDF membrane, gel, filter paper, sponge. The sandwich was clamped shut and placed into the transfer apparatus. The electrodes were connected according to the “red to red, black to black" principle, and the transfer was carried out at 100 V for 75 minutes.

4. Blocking: The PVDF membrane was immersed in TBST blocking solution containing 5% skimmed milk powder and incubated on a shaker at room temperature for 2 hours. For phosphorylated proteins, 1% BSA was used as the blocking solution.

5. The appropriate primary antibodies were diluted in the blocking solution and used to incubate the PVDF membrane overnight at 4°C. The dilution ratios for the primary antibodies were as follows:

Primary antibody name	Dilution ratio
GAPDH	1:10000
HMGCR	1:1000
SQLE	1:1000
ACAT1	1:1000
SREBP2	1:2000
ABCG5	1:1000
ABCG8	1:1000

6. After incubation with primary antibody, the PVDF membrane was washed thoroughly with TBST three times for 10 minutes each time. Subsequently, the HRP-labeled secondary antibody was diluted with TBST at a ratio of 1:3000 to completely immerse the PVDF membrane in the secondary antibody incubation solution. The membrane was incubated on a shaker at room temperature for 2 hours.

7. The enhancer solution A in the ECL reagent was mixed with the stable peroxidase solution B in a 1:1 ratio to prepare the working solution. The PVDF membrane was placed with the protein side facing up, and 200 μL of working solution was evenly dripped onto the membrane from left to right. Subsequently, a chemiluminescence imaging system was used for exposure processing.

8. Grey scale values were analyzed with Image-Pro Plus software.

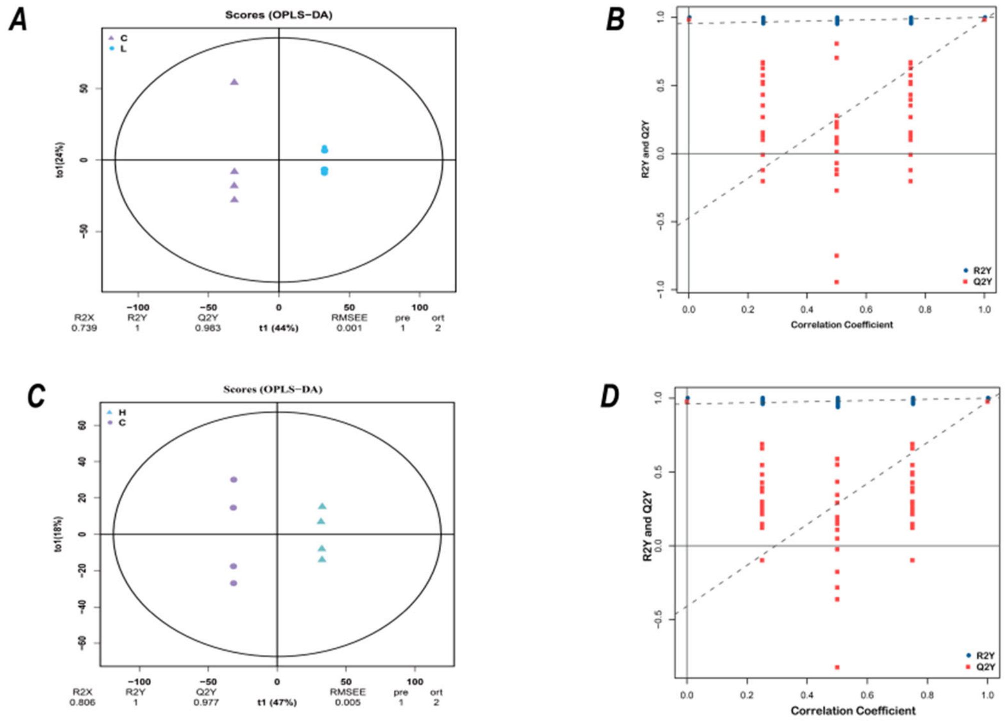

Figure S1. Effects of perinatal exposure to BPS on hepatic off-target metabolomics in F1 female rats. (A, C) OPLS-DA of "Con vs BPS-L" and "Con vs BPS-H". (B, D) permutation test of "Con vs BPS-L" and "Con vs BPS-H".

Table S1. The main kits and antibodies used in the present study.

Kits & Antibodies	Manufacturer	Item No.
Pre-stained protein molecular weight MARKER (10-250KD)	Mei5bio	MF028-plus-01
HMGCR, Rabbit pAb (100KD)	Abclonal	A16875
SQLE, Rabbit pAb (60KD)	Proteintech	12544-1-AP
ACAT1, Rabbit pAb (40KD)	Proteintech	16215-1-AP
SREBP2, Rabbit pAb(124KD)	Affinity	DF7601
ABCG5, Rabbit pAb (73KD)	Boster	PB0381
ABCG8, Rabbit pAb (76KD)	Absin	Abs120422
GAPDH, Rabbit pAb (36KD)	Proteintech	60004-1-Ig
Alanine aminotransferase Assay Kit	Nanjing Jiancheng Bioengineering Institute	C009-2-1
Aspartate aminotransferase Assay Kit	Nanjing Jiancheng Bioengineering Institute	C010-2-1
High-density lipoprotein cholesterol assay kit	Nanjing Jiancheng Bioengineering Institute	A112-1-1
Low-density lipoprotein cholesterol assay kit	Nanjing Jiancheng Bioengineering Institute	A113-1-1
Total cholesterol assay kit	Nanjing Jiancheng Bioengineering Institute	A111-1-1
Rat VLDL ELISA Kit	Jymbio	JYM0815Ra
AFTSpin Tissue/Cell Fast RNA Extraction Kit for Animal	ABclonal	RK30120
ABScript Neo RT Master Mix for qPCR with gDNA Remover	ABclonal	RK20433
2X Universal SYBR Green Fast qPCR Mix	ABclonal	RK21203

Table S2-1: Housekeeping gene stability.

gene	Mean Cq	Coefficient of Variation （CV%）	NormFinder (Stability)
GAPDH	20.9262	2.31	0.54
HPRT1	24.9119	2.35	0.38
TUBULIN	26.4401	2.5	0.47
β-ACTIN	22.5770	2.51	0.30
B2M	20.1488	2.97	0.62

Table S2-2: Original Cq value of housekeeping genes.

housekeeping genes	samples	Con (Cq)	BPS-L (Cq)	BPS-H (Cq)
β-ACTIN	1	22.1311	23.1585	23.9756
	2	22.3485	23.7203	24.9339
	3	22.6188	21.7906	21.665
	4	21.8248	21.7501	22.9572
	5	22.6399	21.8515	22.4765
	6	22.1454	22.4131	21.986
TUBULIN	1	25.9734	27.3477	29.2061
	2	26.7494	26.6972	28.0967
	3	26.6879	25.0601	25.8839
	4	26.5865	25.4113	26.5409
	5	26.6906	25.3408	26.1899
	6	26.2859	25.1873	25.9861
HPRT1	1	24.0358	25.5655	25.9811
	2	23.9614	26.6937	26.5889
	3	25.2548	24.5321	24.5474
	4	24.19	24.9038	25.9587
	5	23.9846	24.8009	24.5237
	6	23.8803	24.6563	24.3543

GAPDH	1	19.7597	25.04	24.2958
	2	20.3858	23.3563	23.4681
	3	20.9419	19.5701	20.2224
	4	19.6497	19.901	21.1647
	5	20.3889	20.5825	19.0768
	6	19.6213	20.6344	18.6116
B2M	1	19.1604	21.3396	21.7035
	2	18.8108	20.4744	21.3467
	3	20.7083	19.8475	20.7074
	4	19.5851	20.3941	18.8374
	5	19.6067	20.4665	20.8733
	6	19.5851	20.3941	18.8374

Table S3. Information for primer-pairs used in SYBR Green-based quantitative real-time PCR assays.

Genes	Primer	Sequence (5'-3')	NCBI
Rat Gapdh	Forward	ACAGCAACAGGGTGGTGGAC	NM_017008.4
	Reverse	TTTGAGGGTGCAGCGAACTT	
Rat Cyp7a1	Forward	ATGTTTCTCAATGACACGCTC	NM_012942.2
	Reverse	CAAGTAAATGGCACTCCCTCC	
Rat Hmgcr	Forward	CATAGGAGGCTACAACGCCC	NM_013134.2
	Reverse	CAGGATTGTCTTTGCACGCC	
Rat Baat	Forward	CCATTGAAAAAGCTCACGGACA	NM_017300.2
	Reverse	AACCTCTCCTCCCCAGTTGA	
Rat Cyp8b1	Forward	GCCGAGACATCCTTCGCTTT	NM_031241.2
	Reverse	CATCAGGGTTGAGGAACCGAT	
Rat Cyp7b1	Forward	AGGTTCTGAGGTTGTGCTCC	NM_019138.1
	Reverse	CTGCCTGGACATTTGCTTGTT	
Rat Acat1	Forward	CGGGCTAACCGATGTCTA	NM_017075.2

	Reverse	GATGGTGATGGGCGTAAT	
Rat Sqle	Forward	GCTTCGGTTACTAGAGG	NM_017136.2
	Reverse	GAGAACTGGACTGGGAT	
Rat Abcg5	Forward	CTTCAGGCAATCGGACAT	NM_053754.2
	Reverse	GTACAACAGCCCTACGC	
Rat Abcg8	Forward	CAAACGGGTGGAAGACG	NM_130414.2
	Reverse	GAGCGGTGAAGCTGTCG	
Rat Srebp2	Forward	CGAGCCTCCCAAAGAAG	NM_001033694.2
	Reverse	TCCTCAGAACGCCAGAC	
Rat Hsd3b7	Forward	GTGGACTTCGGCTGATAGG	NM_001398675.1
	Reverse	GTGCTGACAGTAAAGGTGGTG	

Table S4. Antibody dilution ratio.

Primary antibody name	Dilution ratio
GAPDH	1:10000
HMGCR	1:1000
SQLE	1:1000
ACAT1	1:1000
SREBP2	1:2000
ABCG5	1:1000
ABCG8	1:1000

Table S5. Litter size and female-to-male ratio of F1 offspring.

Group	Litter size	Female-to-male ratio
Con	14.83 ± 0.88	1.19 ± 0.12
BPS-L	12.50 ± 1.20	0.80 ± 0.17
BPS-H	12.00 ± 1.30	0.78 ± 0.20

Note: Litter size was analyzed using n = 12 litters per group; female-to-male ratio was analyzed using n = 6 litters per group. Data are expressed as mean ± SEM. Statistical analysis was performed using one-way ANOVA; no significant differences were observed among groups ($P > 0.05$). These

data were originally published in our previous study [1] and are included here for completeness.

Reference:

[1] Zhao, M.; Xie, Y.; Xu, X.; Zhang, Z.; Shen, C.; Chen, X.; Zhu, B.; Yang, L.; Zhou, B. Reproductive and transgenerational toxicity of bisphenol S exposure in pregnant rats: Insights into hormonal imbalance and steroid biosynthesis pathway disruption. Sci. Total Environ. 2024, 927, 172379. https://doi.org/10.1016/j.scitotenv.2024.172379.
